# Improving the parameters of electron transport in quantum dot sensitized solar cells through seed layer deposition†

**DOI:** 10.1039/c8ra04413a

**Published:** 2018-07-19

**Authors:** Mahmoud Samadpour

**Affiliations:** Department of Physics, K. N. Toosi University of Technology PO Box 15418-49611 Tehran Iran samadpour@kntu.ac.ir

## Abstract

Here we investigate the effect of seed layer deposition on electron-transport parameters of chemical-bath-deposited (CBD) CdSe quantum dot sensitized solar cells (QDSCs). Fill factors were systematically improved to more than 0.6 through reduced recombination after seed layer deposition. Considering the beneficial effects of seed layer deposition, noticeably higher efficiency values were systematically obtained in cells with the seed layer (2–3.19%) in comparison to cells without a seed layer (0.03–0.46%) depending on the TiO_2_ photoanode particle size. Electron-transport parameters in cells, including chemical capacitance, recombination resistance, the diffusion coefficient, electron life time and small perturbation diffusion lengths of electrons were examined by modeling the experimental impedance spectroscopy data. We showed that a seed layer enhanced recombination resistance in cells, while the photoanode conduction band position was not affected. Higher diffusion lengths of electrons were obtained after seed layer deposition, correlated to the reduced electron recombination rate by redox electrolyte through seed layer deposition. As a general conclusion we report that while the seed layer generally is deposited to increase light absorption, at the same time this could be applied in order to systematically enhance charge-transport properties in cells and it has a clear application in the optimization of QDSC performance.

## Abstract 2

It is proved that the seed layer deposition could be systematically applied in order to enhance the charge transport in the cells.

## Graphical abstract

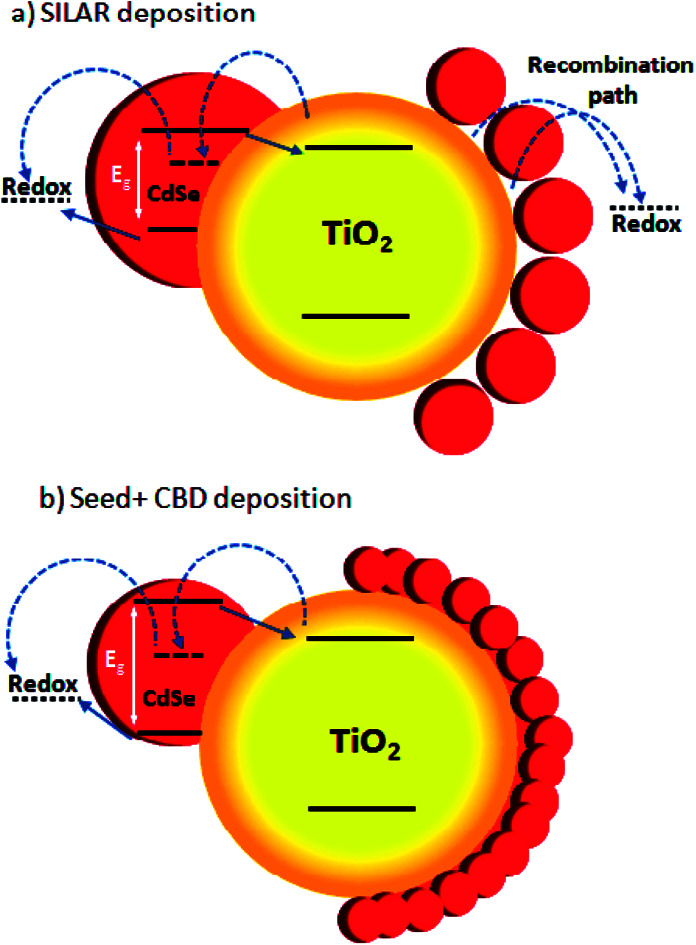

## 1. Introduction

In the past decade, considerable research has been conducted to improve the performance of dye and quantum-dot sensitized solar cells (DSSCs and QDSCs, respectively).^1–12^ In DSSCs and QDSCs, the TiO_2_ photoanode is sensitized by dye/semiconductor quantum dots (QDs) and a redox electrolyte transports charge carriers between the photoanode and cathode electrode.^13–16^ Currently different dye molecules are developed by various researchers^17–22^ and some of them are produced in a large scale by various companies. Dye molecules are well adsorbed as a monolayer on the TiO_2_ surface by their designed anchoring groups and their photogenerated electrons are injected into the TiO_2_ conduction band (CB) with an almost 100% efficiency.^14,23,24^ In comparison to dyes, semiconductor QDs have interesting properties like: tunable light absorption properties by controlling their size/shape; multiple exciton generation by a single photon; simple fabrication methods and low prices.^5,23,25^ The theoretical prediction of possibly more than 30% efficiencies in QDSCs^5,26^ and the mentioned interesting properties motivated many studies to enhance the photovoltaic properties of QDSCs. In spite of the similarities between DSSCs and QDSCs in structure and working principles, replacing dye molecules with semiconductor QDs has raised numerous challenges to the development of QDSCs. In comparison to dyes, adsorption of semiconductor QDs on the TiO_2_ surface encounters some challenges. So far, various methods have been used to deposit QDs on the photoanode structure. For example *ex situ* methods such as direct adsorption of pre-synthesized QDs on TiO_2_ surface and assisted linker molecules have been employed by researchers.^13,23,27^ These methods suffer from the low deposition of QDs and charge transfer distortion by linker molecules, respectively. Currently *in situ* methods like CBD and successive ionic layer adsorption and reaction (SILAR) are generally used for deposition of QDs in QDSCs.^5,28–34^ In these methods, QDs size and thickness of the deposited layer are simply adjusted by controlling synthesizing parameters, *e.g.* temperature of chemical bath, deposition time, number of SILAR cycles and the precursor's molarity. In spite of the superior properties of *in situ* methods compared to *ex situ* ones, they produce multi-layers of deposited QDs on TiO_2_ surface while a monolayer of dye molecules is adsorbed in DSSCs.^23,34–37^ This means that some parts of semiconductor QDs are not in a direct contact with TiO_2_ and, consequently, the photogenerated electrons in these QDs should pass through their neighbors before reaching to TiO_2_ conduction band. The mentioned interlayer transport could disturb charge transport in cells as QDs have a considerable density of deep and surface trap states with different energy levels.^23,35,36^ It is important to note that, in DSSCs, this is not the case since dye molecules have clear HUMO and LUMO energy levels and are adsorbed as a monolayer on TiO_2_ surface. Currently various dye molecules with specific absorption spectrum and HUMO and LUMO energy levels are used in DSSCs without concerning about trap states or hopping transport between dyes.^17,18,21,22^ Although we easily adjust light absorption by controlling the synthesizing parameters in SILAR/CBD deposition methods, we change the density of surface trap states and the energy band structure of QDs. Consequently, the charge transport properties of the cell will definitely change and, therefore, their photovoltaic properties are determined by light absorption and the charge transport properties. While considerable research has been conducted to increase light absorption by various deposition methods, the influence of the mentioned methods on the charge properties of cells is not considered in a clear way.^28,38,39^ Reviewing the literature shows that a seed layer is generally deposited on TiO_2_ surface before CBD deposition.^3,33,40,41^ The seed layer increases the sensitizer layer thickness and, consequently, increases light absorption in the cell. For instance, CdSe QDs were deposited as a seed layer by Fan *et al.*^3^ and efficiency was improved to 3.21% in comparison to 1.46% which was obtained without the seed layer. Also, a 4.94% efficiency was achieved in ZnSe/CdS/CdSe sensitized cells,^42^ recently improved to 7.24% by modifying the structure to ZnSe/CdS/CdSe/ZnSe sensitized cells.^40^ In both structures, the first ZnSe layer was deposited as a seed layer which considerably enhanced the amount of CdS/CdSe deposition and thus light harvesting. Moreover, a CdS layer was deposited by a self-assembly method as a seed layer before CdSe sensitization by Lee *et al.*^38^ They showed that the seed layer facilitates the nucleation and growth of CdSe QDs. Furthermore, the effect of SILAR and seed layer/CBD methods on the photovoltaic properties of cells was investigated by Zhou *et al.*^43^ They reported that CBD method improves recombination resistance in cells and a 4.85% efficiency was obtained by optimizing the deposition parameters.^43^

They concluded that the CBD method has superior properties compared to the SILAR method for the conformal coverage of QDs on the TiO_2_ substrate, while the parameters of electron transport in cells, *e.g.* diffusion coefficient, photoanode conduction band shift and small perturbation diffusion lengths of electrons were not explored.

Recently, the effect of linker seeding before chemical bath deposition was studied by Yan *et al.*^41^ They demonstrated that linker seeding enhances the open circuit voltage and fill factor of cells. Their results indicated that linker seeding reduces charge recombination in cells. In spite of these interesting results, linker seeding is performed under argon atmosphere, a method which is not easy to perform and not generally used in QDSCs. Also, the photoanode was made by TiO_2_ microspheres which are a special structure not normally used in QDSCs. It must be noted that the photoanode structure can strongly change the charge-transport properties of the cell in the same way as the linker seed layer.^33^ As a result, a systematic study on various photoanode structures is needed in order to attain a general conclusion about the effect of seed layer on the charge-transport properties of cells. Here for the first time we have investigated the effect of TiO_2_ support morphology on the results of CdSe seeding before the further deposition by CBD method. In the present study, we investigated the effect of conventional seed layer deposition on the charge properties of cells in a systematic way. Here, we also examined the effect of seed layer on the light absorption properties of cells while simultaneously exploring the effect of a seed layer on electron-transport parameters in detail. In this study, various parameters such as chemical capacitance, recombination resistance, diffusion coefficient, electron life times and electrons diffusion lengths were studied in a systematic way. We show that the seed layer increases recombination resistance in cells while the photoanode conduction band position is not affected. Higher electron diffusion lengths were obtained after seed layer deposition which is correlated to the reduced electron recombination rate by redox electrolyte through seed layer deposition.

Our results indicate that, contrary to DSSCs, all electron-transport parameters should be reconsidered after any changes are made in the QDs' deposition parameters to increase light absorption.

## 2. Experimental section

### 2.1. Preparation of the TiO_2_ structures

Here three different commercial TiO_2_ pastes containing: DSL-18NR-T from Dyesol company (particle size 20 nm), WER 2-O (particle size 200 nm) from Dyesol company and PST-400 (particle size 20–400 nm) from Sharif Solar company, were used in order to make various photoanode structures. We name these pastes S20, S200 and S400 respectively. All photoanodes were prepared by the deposition of two layers on the fluorine doped tin oxide (FTO) glass substrates by the dr blade method. Photoanodes were annealed at 450 °C for 30 minutes before sensitization by semiconductor QDs. Three different TiO_2_ structures were made and tested here. These structures have various particle sizes and consequently produce photoanodes with clearly different effective surface areas.

### 2.2. Photoanode sensitization

All TiO_2_ photoelectrodes were sensitized by CdSe QDs with *in situ* deposition methods. CdSe deposition was performed by SILAR/CBD methods. The SILAR process was carried out following the method developed before.^44^ For the SILAR deposition, a 0.03 M Cd(NO_3_)_2_ in ethanol was prepared as the Cd^2+^ precursor. Also a 0.03 M Se^2−^ in ethanol was utilized as the Se^2−^ precursor. In order to prepare the Se^2−^ precursor solution, SeO_2_ was reduced by NaBH_4_ in ethanol under N_2_ atmosphere. During the stirring, the red color of the solution was changed to the transparent after about 20 minutes, indicating the reduction of SeO_2_. The solution was then transferred into a glove box under N_2_ atmosphere in order to perform the SILAR sensitization.

For the each CdSe SILAR cycle, TiO_2_ electrode was dipped into the Cd^2+^ precursor for 30 second and subsequently into the Se^2−^ precursor. After each Cd^2+^ or Se^2−^ precursor bath, anodes were rinsed by ethanol and subsequently were dried by an Ar gun. Here, 7 SILAR cycles is performed for sensitization of the TiO_2_ electrodes. The CBD was carried out according to the method which described before:^6,7^ 80 mM Na_2_SeSO_3_ solution was made by refluxing Se and Na_2_SO_3_ powders in Milli-Q water at 70 °C for 3 hours under N_2_ atmosphere. The aqueous chemical bath solution was prepared by mixing the 120 mM nitrilotriacetic acid with 80 mM of CdSO_4_ and Na_2_SeSO_3_. TiO_2_ electrodes were immersed in the chemical bath solution at 10 °C. After 18 hours of the chemical bath deposition, electrodes were rinsed by the Milli-Q water and dried with Ar pressure. In some other samples before the CBD deposition, a CdSe seed layer was deposited on the TiO_2_ structures by the SILAR method. The SILAR method was same as explained before. It is well known that the seed layer significantly enhances the growth rate of CdSe QDs. This could potentially enhance the light absorption for the same CBD deposition time. After the CdSe deposition by the SILAR/CBD methods, all the samples were coated by ZnS QDs in order to improve the cell's stability and also reducing the charge recombination. Here ZnS was deposited by dipping the CdSe sensitized photoanodes into the 0.1 M Zn(CH_3_COO)_2_ and 0.1 M Na_2_S solutions according the method that we explained recently.^7^

### 2.3. QDSC preparation and characterization

Polysulfide electrolyte was prepared by dissolving 2 M Na_2_S and S powders in Milli-Q ultrapure water under stirring for about 45 minutes. In order to make Cu_2_S CEs, brass substrates were dipped in the HCl solution at 80 °C for 10 min and subsequently were rinsed by the DI water and dried by an Ar gun. Finally a drop of the polysulfide electrolyte was put on the modified brass substrate which led to the preparation of the Cu_2_S electrode. Solar cells were prepared by sandwiching photoanodes and CEs. Here scotch tapes with 50 μm thickness is applied as a spacer.

### 2.4. Photoanode and solar cell characterization methods

Surface area measurements were performed according to the Brunauer–Emmett–Teller (BET) method by Quanta Sorb machine. Morphology of the TiO_2_ structures was investigated by field emission scanning electron microscopy (FESEM). The optical absorption and reflection spectra of the photoanodes were recorded by a Cary 500 UV-VIS spectrometer from Varian company. Current–potential (*J*–*V*) curves, were measure by *I*–*V* tracer device from the Sharif Solar company. Impedance spectroscopy (IS) and applied bias voltage decay (ABVD) measurements were measured with a PGSTAT-30 potentiostat from Autolab Company. Impedance spectroscopy measurements were carried out in the dark condition. Here frequency was swiped between 400 kHz to 0.1 Hz at various forward biases. Cells were illuminated using a solar simulator (Sharif Solar Company) at AM 1.5G under 100 mW cm^−2^ light intensity. Incident photon to electron conversion efficiency (IPCE) measurements is carried out by a monochromator while the photocurrent is measured using a nanoammeter.

## 3. Results and discussion

**Fig. 1:**
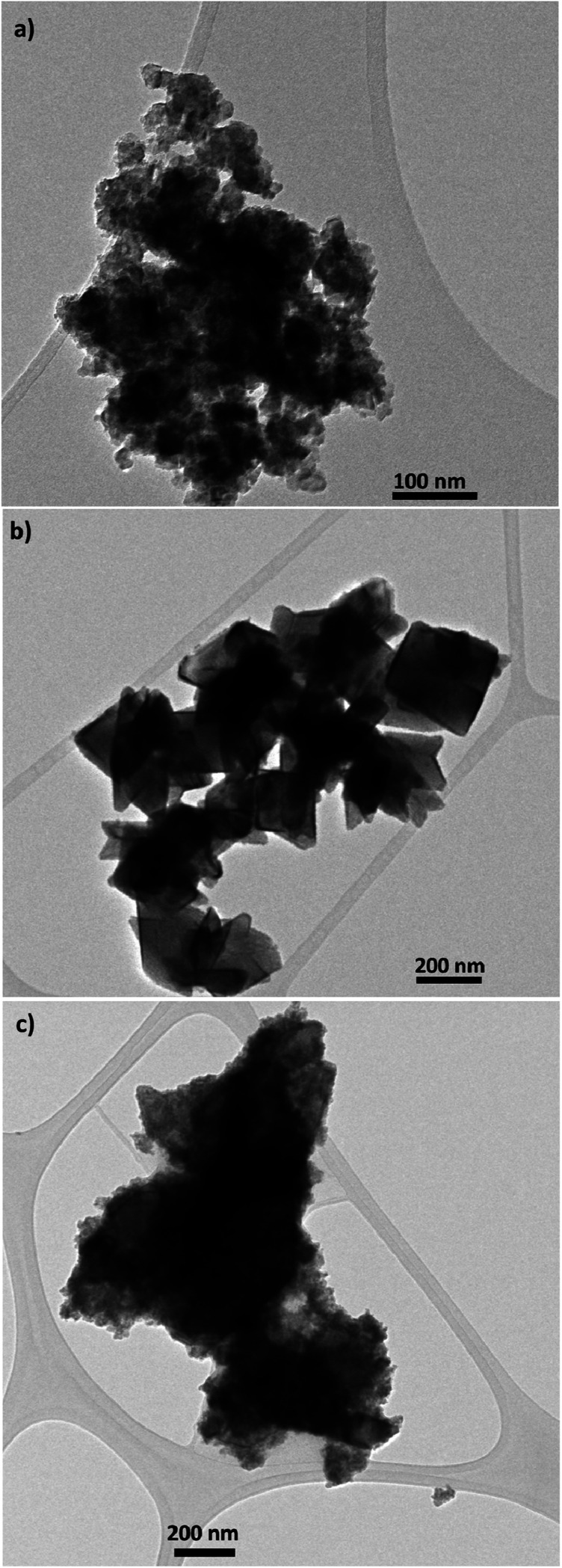
TEM micrograph of (a) S20, (b) S200 and (c) S400 pastes, scale bar is 100 nm in (a), and 200 nm in (b) and (c).

**Fig. 2:**
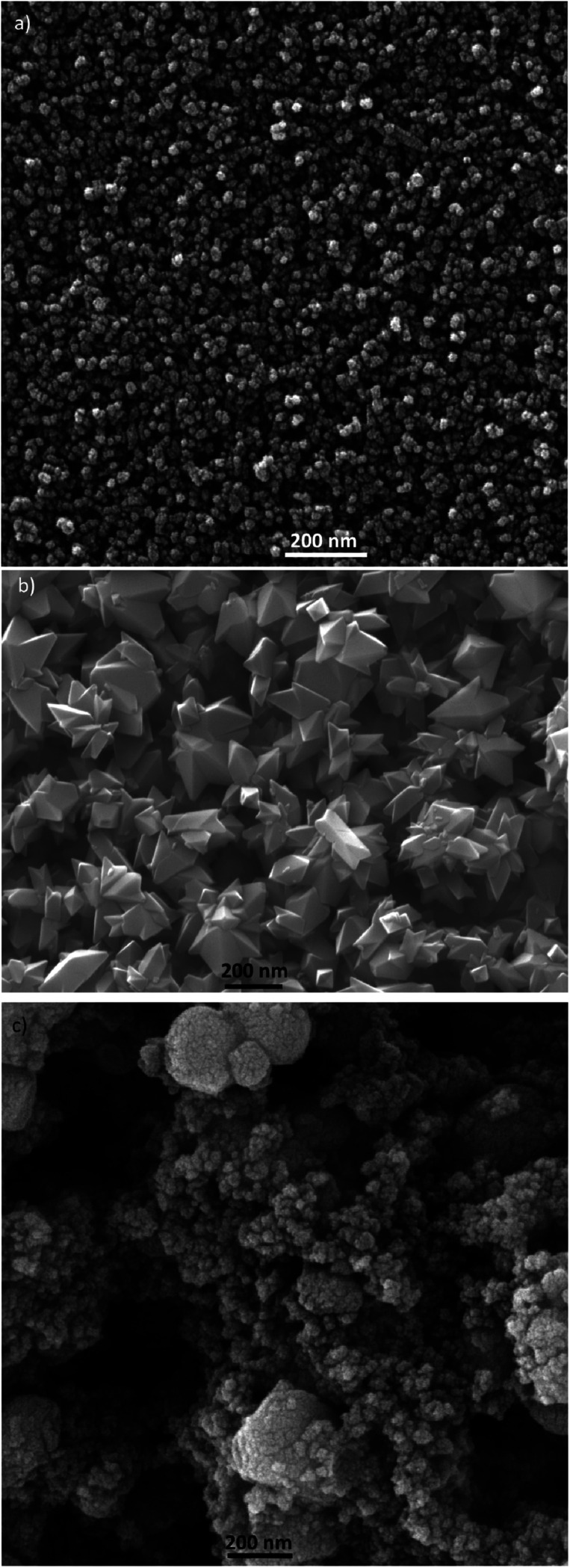
SEM micrograph of (a) S20, (b) S200 and (c) S400 pastes, scale bar is 200 nm in all figures.

TEM and SEM micrograph of S20, S200 and S400 pastes are presented in Fig. 1 and 2 respectively.

Based on Fig. 1, TiO_2_ pastes have nanoparticulated structures with different morphologies. Also a distribution of sizes could be seen in S400 structures. According to Fig. 2, the S20 paste contained an almost 20–30 nm particle size which provides a high surface area for the deposition of CdSe QD sensitizers. The S200 paste was made by an approximately 200 nm particle size which could potentially decrease the amount of QDs' loading in the photoanode. According to Fig. 2c, the S400 paste is made by a wide range of particle sizes from 20 to 400 nm. Therefore, this layer not only could have an adequate surface area but also can be used as an effective light-scattering layer in QDSCs.

**Fig. 3:**
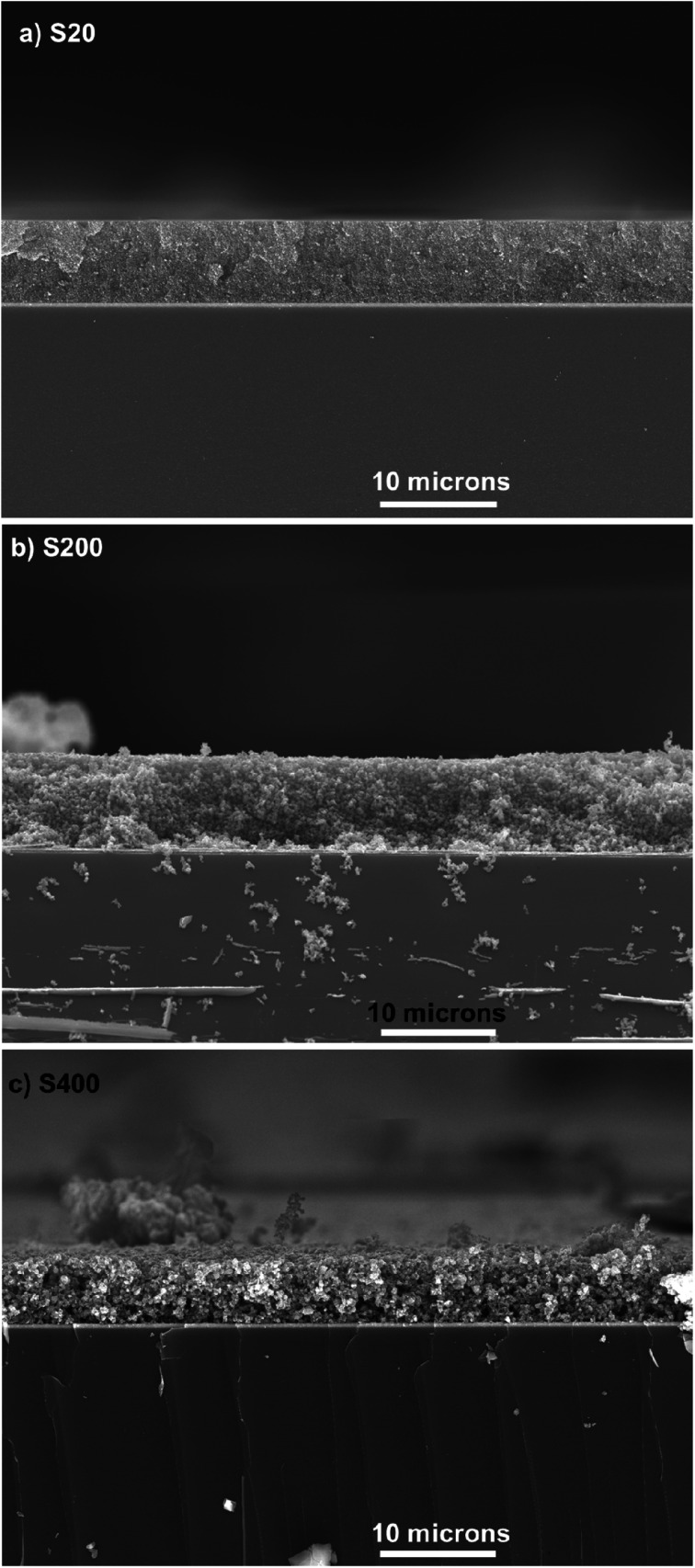
Cross section SEM micrograph of (a) S20, (b) S200 and (c) S400 samples, scale bar is 10 microns in all figures.

The thickness of TiO_2_ layers was explored by the cross-section SEM. Fig. 3 indicates the cross-section SEM micrograph from S20, S200, and S400 samples.

**Fig. 4:**
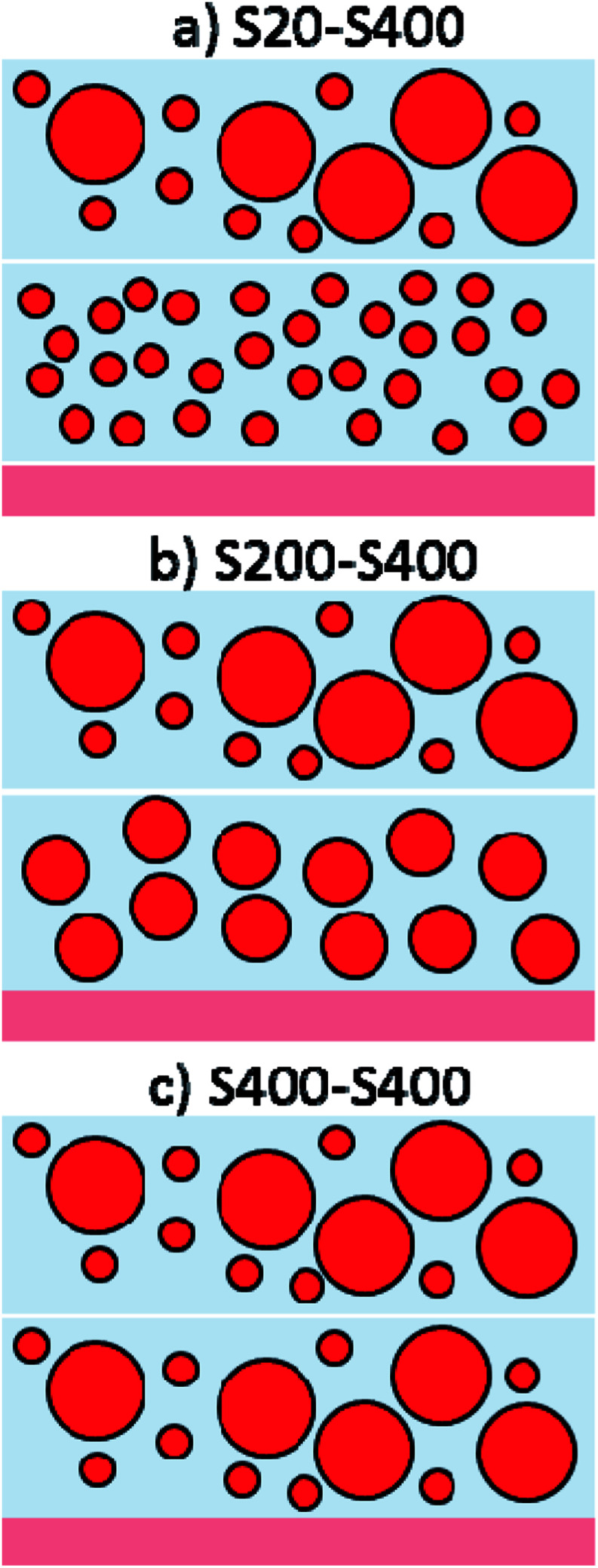
Three different photoanode structures are made by S20, S200 and S400 pastes containing: (a) S20-S400, (b) S200-S400 and (c) S400-S400 structures.

According to these figures, S20, S200, and S400 structures have a thickness of 7, 7.5, and 5.5 μm, respectively. Three different photoanode structures were made by these pastes, including S20-S400, S200-S400, and S400-S400 (Fig. 4).

Each structure was made by the dr blade deposition of two pastes on the FTO substrate. For example, S20-S400 (Fig. 4a) means that the first layer is deposited from the S20 paste (Fig. 2a) and the second layer is made by the S400 paste (Fig. 2c).

More SEM micrograph of S20, S200, S400, S20-S400, S200-S400 and S400-S400 structures is presented in ESI (Fig. S1†).

Here, we examined various TiO_2_ structures in order to conduct a systematic study on the effect of seed layer on electron transport properties in QDSCs. The thickness of S20-S400, S200-S400, and S400-S400 was 12 ± 1, 13 ± 1, and 11 ± 1 μm, respectively, based on profilometry measurements. It is important to note that the photoanodes structure could affect the electron-transport parameters in cells in a clear way. The effect of photoanodes structure (thickness, surface area, and pore size) on the cells' performance was previously explained by us^33^ and also by Zhang *et al.* in detail.^45^ Regarding the crucial role of the photoanode structure, here we compared the effect of seed layer deposition on the cells with the same structure. For example, we have investigated S20-S400 structures with and without a pre deposited CdSe seed layer. We also conducted a systematic study on various structures (S20-S400, S200-S400, and S400-S400) in order to make sure that our study on the seed layer is not affected by a specific structure.

**Fig. 5:**
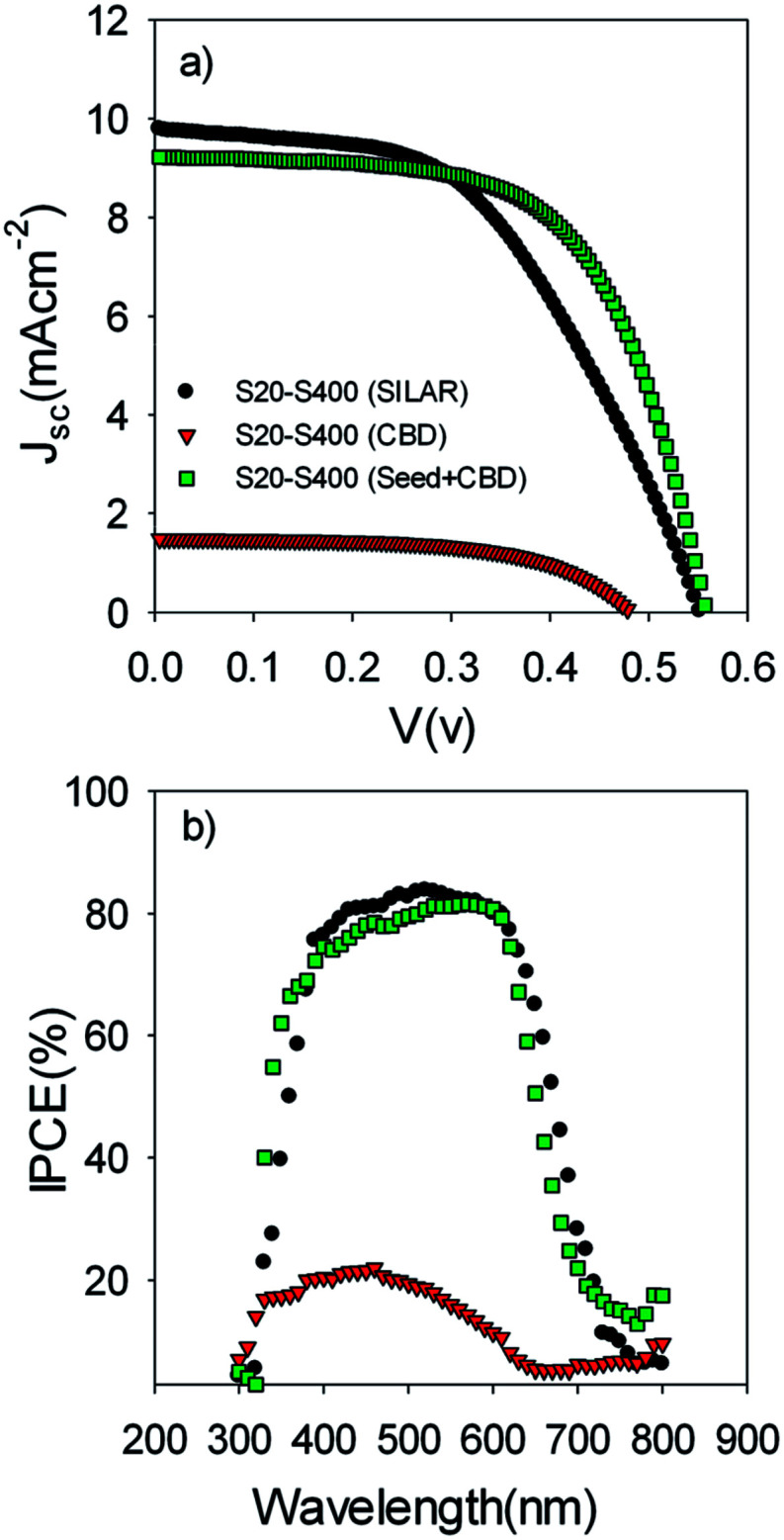
(a) Current voltage and (b) IPCE properties of the S20-S400 cells which are sensitized by SILAR, CBD and SILAR (3 cycles of CdSe as a seed layer)/CBD (Seed + CBD) method.

Fig. 5a illustrates the current voltage properties of S20-S400 cells which were sensitized by the SILAR method (7 cycles of CdSe), CBD method, and SILAR (3 cycles of CdSe as a seed layer)/CBD method. We named these cells S20-S400 (SILAR), S20-S400 (CBD), and S20-S400 (Seed + CBD) for an easier discussion.

The corresponding photovoltaic parameters of Fig. 5a are indicated in Table 1 for a clear examination.

**Table 1:** Photovoltaic parameters of the QDSCs: photocurrent *J*_sc_, open circuit voltage *V*_oc_, fill factor FF, and efficiency *E*, as a function of the sensitization method tested under standard AM 1.5G conditions

Anode type	*V*_oc_ (mV)	*J*_sc_ (mA cm^−2^)	FF	*E* (%)
S20-S400 (SILAR)	550 ± 9	9.78 ± 0.06	0.53 ± 0.01	2.86 ± 0.11
S20-S400 (CBD)	477 ± 27	1.50 ± 0.12	0.55 ± 0.02	0.40 ± 0.06
S20-S400 (Seed + CBD)	555 ± 11	9.25 ± 0.07	0.62 ± 0.01	3.19 ± 0.13

**Fig. 6:**
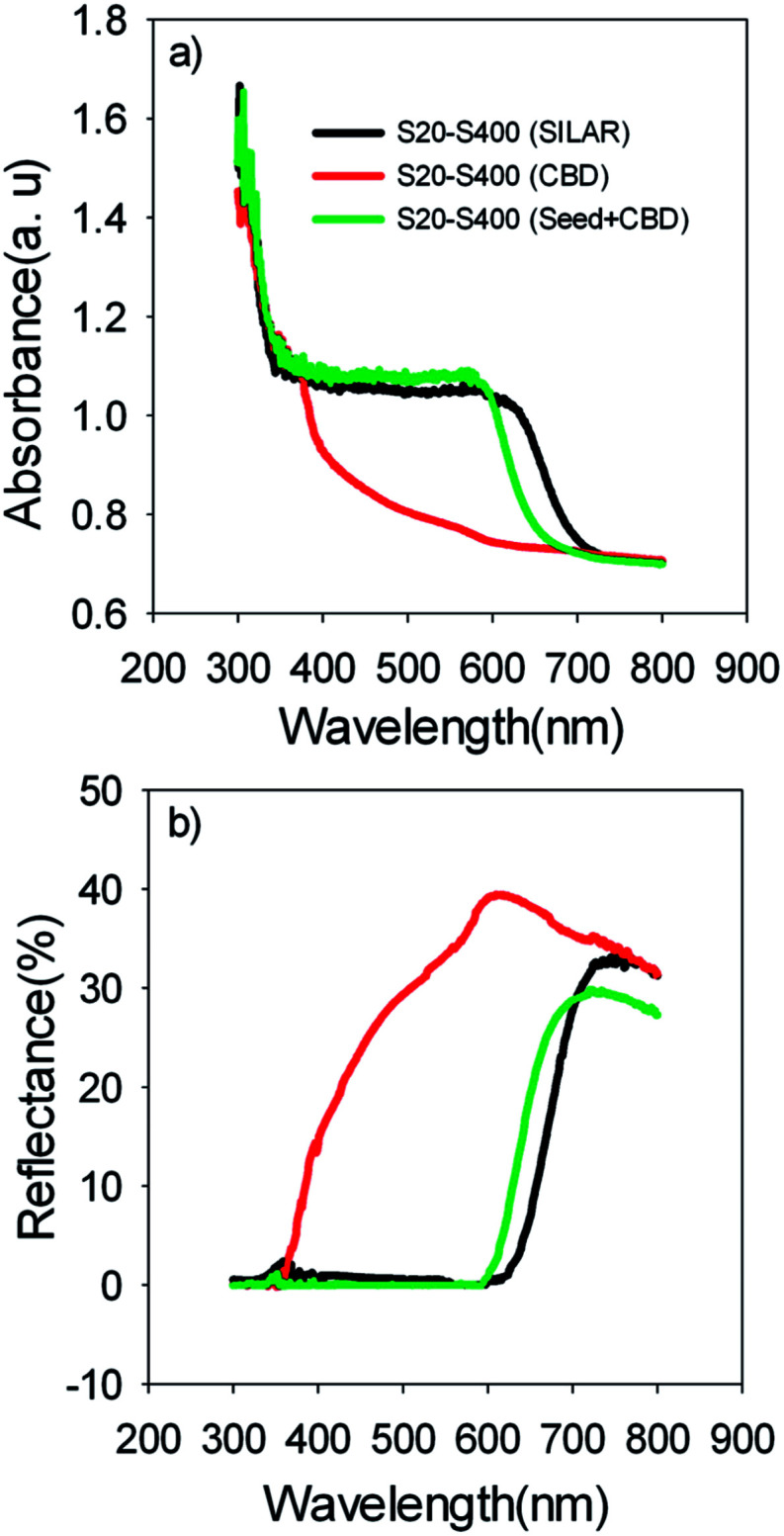
(a) Absorption and (b) reflection spectrum of the S20-S400 cells which are sensitized by SILAR, CBD and Seed + CBD method.

Based on Table 1, S20-S400 (CBD) structures show considerably lower *J*_sc_ values (1.50 mA cm^−2^) in comparison to the other structures (more than 9 mA cm^−2^). This can be explained by considering the results in the Fig. 6a. According to this figure, the light absorption in S20-S400 (CBD) structure is considerably lower than S20-S400 (SILAR), and S20-S400 (Seed + CBD) structures, indicating the very low deposition of QDs on TiO_2_ structure. The lower adsorption of QDs in S20-S400 (CBD) structures was also clearly observed from the comparison of photoanodes' color by the naked eye. The low deposition of QDs in S20-S400 (CBD) structures decreases the light harvesting in the cells. Therefore, the rate of electron–hole generation by the incident light decreases and lower *V*_oc_ and *J*_sc_ values could be expected.

Fig. 5b indicates the corresponding IPCE values of cells in Fig. 5a. According to Fig. 5b, a clear red shift was seen in IPCE values for SILAR-sensitized cells in comparison to cells with the seed layer. Thus, a higher current density was obtained for SILAR sensitized cells (Table 1).

Fig. 6b indicates the diffuse reflection of cells. Based on Fig. 6a and b, a clear red shift in both absorption and reflection spectrum is seen in SILAR sensitized cells in comparison to cells with the seed layer.

The size of the CdSe QDs was estimated using eqn 1^46,47^1
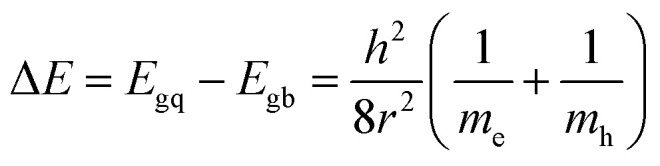
where Δ*E* is the band gap shift, *E*_gq_ is the band gap of quantum dots; *E*_gb_ is the band gap of the bulk material (1.74 eV for CdSe), *h* is the Planck constant, *r* is the QD radius, *m*_e_ and *m*_h_ are the effective masses of electron and hole respectively (*m*_e_ and *m*_h_ of the CdSe are 0.13 *m*_0_ and 0.44 *m*_0_ respectively, *m*_0_ = 9.11 × 10^−31^ kg). Optical band gap of CdSe QDs was obtained from the absorption band edges in the absorption spectrum (Fig. 6) as previously explained.^48^ Optical band gap of S20-S400 (SILAR), S20-S400 (Seed + CBD), and S20-S400 (CBD) was obtained 1.84, 1.93, and 3.02 eV respectively.

From the eqn (1), size of CdSe QDs was obtained 7.86, 5.51, and 2.16 nm for S20-S400 (SILAR), S20-S400 (Seed + CBD), and S20-S400 (CBD) respectively.

**Fig. 7:**
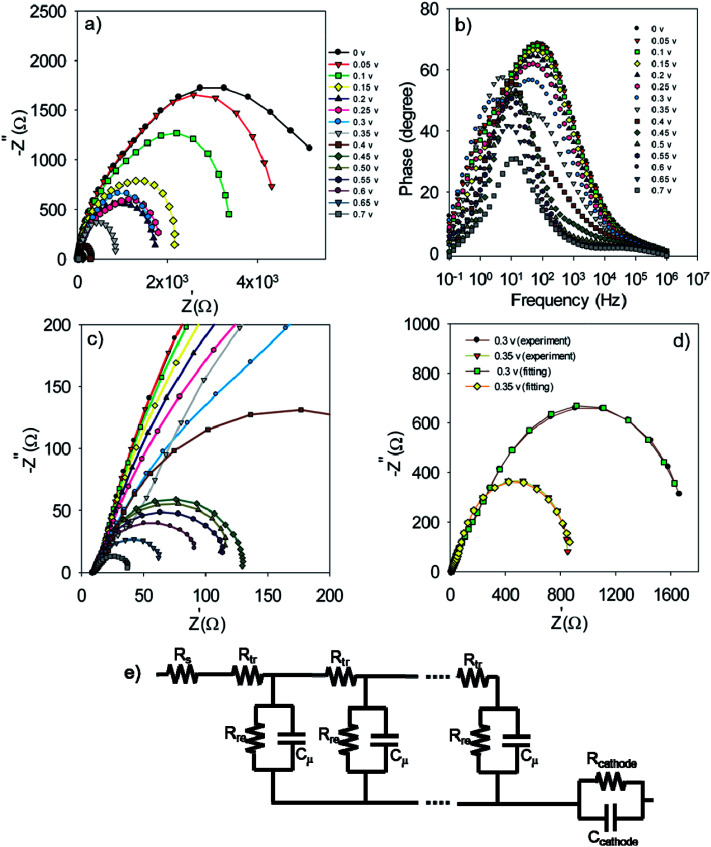
Nyquist (a and c) and Bode plot (b) for S20-S400 (SILAR) structures. Typical fitting results for the experimental impedance data at 0.3 and 0.35 volt bias voltages (d). (e) The model which is used for the fitting. Here *R*_s_, *R*_tr_, *R*_re_, *R*_cathode_, *C*_μ_ and *C*_cathode_ are the series resistance, electron transport resistance in the photoanode, electron recombination resistance at the photoanode/electrolyte interface, charge transfer resistance at the cathode/electrolyte interface, chemical capacitance of the photoanode and the cathode chemical capacitance, respectively.

This result proves that CdSe QDs have a larger size in SILAR sensitized cells in comparison to cells which are sensitized by the Seed + CBD method. In other words, the size of QDs is further confined in cells with seed layers in comparison to SILAR-sensitized cells. The red shift observed in the absorption spectrum was in good agreement with the red shift in IPCE results (Fig. 5b) as previously explained. According to Table 1, in spite of enhanced current densities in SILAR sensitized cells, fill factor (0.53 *vs.* 0.62) is reduced compared with Seed + CBD sensitized cells. This could be explained by the enhanced charge recombination resistance of S20-S400 (Seed + CBD) structures in comparison to S20-S400 (SILAR) structures.^41,43^ Charge recombination resistance was obtained from impedance spectroscopy results which will be explained in more detail in Fig. 7. Consequently, lower efficiencies were achieved in SILAR sensitized cells (2.86%) in comparison to cells with the seed layer (3.19%). Also the stability of the cells was investigated under light-exposure. The photovoltaic parameters of QDSCs containing: *V*_oc_, *J*_sc_, fill factor, and conversion efficiency (*E*) are shown in Table 2. Based on Table 2, the as prepared cells showed an energy conversion efficiency of 3.22% which decreased to 2.79% after one day. Our results indicate that near 85% of the cells' efficiency is obtained after 3 days which is comparable with the stable cells which were explained before.^49^

**Table 2:** Photovoltaic parameters of the QDSCs: photocurrent *J*_sc_, open circuit voltage *V*_oc_, fill factor FF, and efficiency *E*, as a function of light-exposure times, tested under standard AM 1.5G conditions

Light-exposure time (h)	*V*_oc_ (mV)	*J*_sc_ (mA cm^−2^)	FF	*E* (%)
As prepared	558	9.33	0.62	3.22
24	551	8.89	0.57	2.79
48	554	8.25	0.60	2.74
72	563	7.87	0.61	2.70

It is noteworthy that the S20-S400 photoanode (Fig. 4a) is a general structure normally utilized in DSSCs and QDSCs.^19,36,50^ According to the literature, photoanode structures are generally made by a small-size transparent layer deposited on the FTO substrate (S20) and the second layer is manufactured by larger particle sizes (S400) in order to back scatter the incident light into the cell and improve its efficiency. Here, in S200-S400 (Fig. 4b) and S400-S400 (Fig. 4c) structures, the first layer is not transparent and contains 200 and 400 nm particle sizes, respectively, which have scattering properties. As a result, some parts of the incident light could be scattered before entering the cells, decreasing the efficiency. Although these structures are not common photoanode structures, they were investigated here in order to conduct a systematic study on the effect the seed layer on cell's performance while removing the effect of the photoanode structure.^33^

The current voltage properties of S200-S400 cells which were sensitized by SILAR, CBD and Seed + CBD methods are presented in the ESI (Fig. S2 and Table S1†). We refer to these cells as S200-S400 (SILAR), S200-S400 (CBD), and S200-S400 (Seed + CBD) for ease of discussion.

Here also SILAR sensitized cells presented higher current densities than CBD sensitized ones while their open circuit voltage is less (Fig. S2†). A clear red shift was observed in IPCE values for SILAR sensitized cells (Fig. S2b†), proving that CdSe QDs have a larger size in SILAR sensitized cells in comparison to cells which are sensitized by the Seed + CBD method. More discussion on photovoltaic properties of the S200-S400 structures is presented at ESI.† The current voltage properties of the S400-S400 (SILAR), S400-S400 (CBD), and S400-S400 (Seed + CBD) cells are indicated in Fig. S3 and Table S2.†

Our results showed that SILAR sensitized cells have higher current densities than CBD sensitized ones, while their open circuit voltage and fill factor is lower (Table S2†). Comparison of the results in Tables 1, S1, and S2† suggests that the same systematic photovoltaic properties are presented during SILAR, CBD, and Seed + CBD deposition, unaffected by the photoanode structure (S20-S400, S200-S400, or S400-S400), although these structures have completely different surface areas and particle size distributions. These results indicate that seed layer deposition can be introduced as a systematic method to enhance the photovoltaic properties of QDSCs regardless of photoanode structure details.

In order to obtain more detailed information on the photoelectrochemical performance of cells, impedance spectroscopy was performed in dark condition at various forward biases. The Nyquist and Bode plots for the S20-S400 (SILAR) structures are represented in Fig. 7a, c and b respectively.

**Fig. 8:**
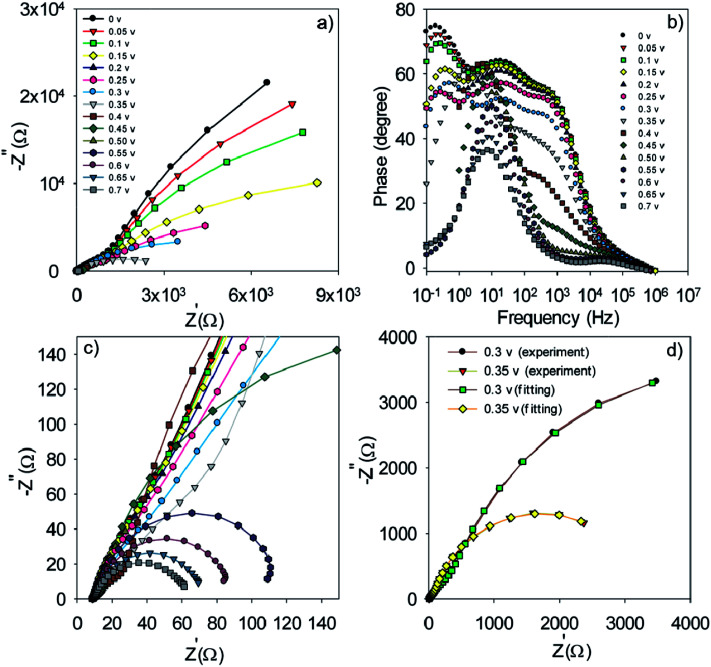
Nyquist (a and c) and Bode plot (b) for the S20-S400 (Seed + CBD) structures. Typical fitting results for the experimental impedance data at 0.3 and 0.35 volt bias voltages (d).

The experimental data is fitted by the previously explained model t (Fig. 7e)^51^ and various electron-transport parameters such as chemical capacitance; *C*_μ_, and recombination resistance; *R*_rec_, are extracted from fitting results. Fig. 7d illustrates the typical fitting results obtained for experimental impedance data at 0.3 and 0.35 V bias voltages. According to this figure, the experimental data are fitted by the proposed model (Fig. 7e) in a clear manner. Here *R*_s_, *R*_tr_, *R*_re_, *R*_cathode_, *C*_μ_ and *C*_cathode_ are the series resistance, electron transport resistance in the photoanode, electron recombination resistance at the photoanode/electrolyte interface, charge transfer resistance at the cathode/electrolyte interface, chemical capacitance of the photoanode and the cathode chemical capacitance, respectively (Fig. 7e). Impedance spectroscopy measurement was also performed for S20-S400 (Seed + CBD) structures, and the Nyquist and Bode plots are represented in Fig. 8a, c and b, respectively. Fig. 8d presents the typical fitting results for experimental impedance data at 0.3 and 0.35 V bias voltages. Here, too, the results were fitted by the model explained before in Fig. 7e.

**Fig. 9:**
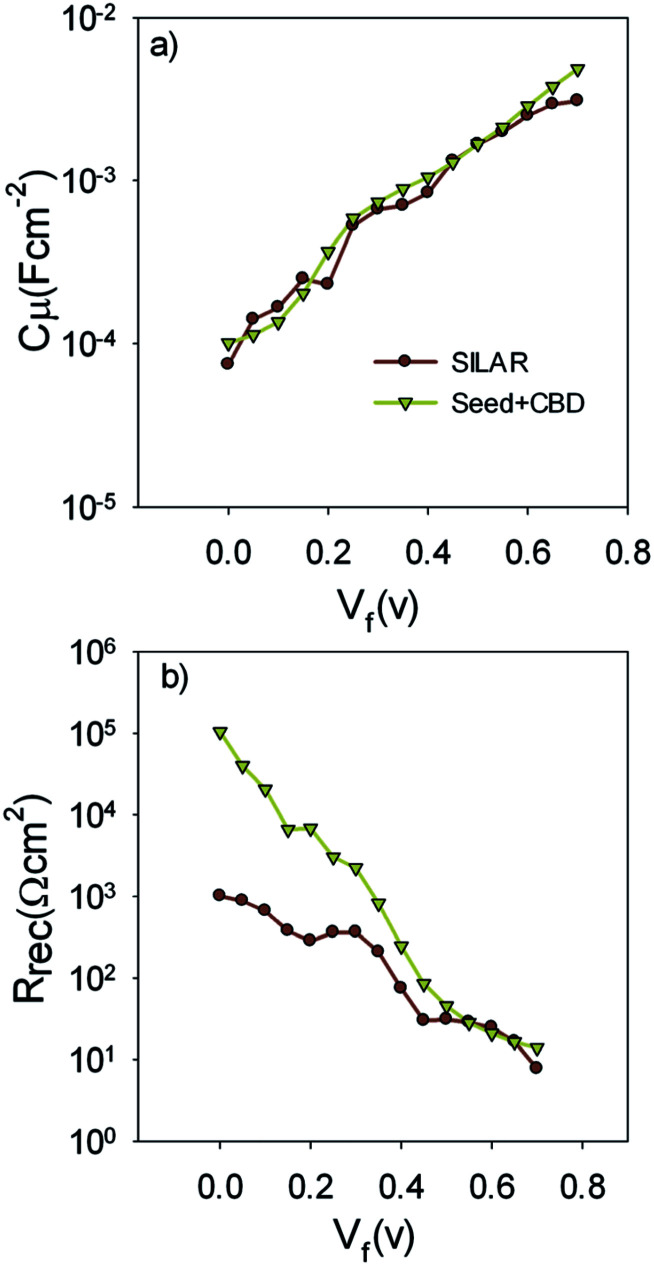
Chemical capacitance; *C*_μ_ and recombination resistance; *R*_rec_ are extracted from the fitting results and are plotted *versus* the voltage drop at the TiO_2_ (*V*_F_).

Fig. 9a indicates the chemical capacitance of S20-S400 (SILAR) and S20-S400 (Seed + CBD) solar cells *versus* the voltage drop at TiO_2_ (*V*_F_).

Based on Fig. 9a, chemical capacitance, *C*_μ_, has an exponential behavior *versus* voltage drop at TiO_2_ (*V*_F_). Consequently, an exponential distribution of trap states is expected near the conduction band edge.^36,51,52^ From this figure, it is clear that the chemical capacitance of SILAR sensitized cells is very similar to that of cells with the seed layer. Therefore, the deposition of seed layer did not affect the relative position of the TiO_2_ conduction band.^52,53^ This result indicates that the higher *V*_oc_ values in cells with the seed layer (Tables 1, S1, and S2†) are not originated from the TiO_2_ CB position. These results, approves the same band structure of the TiO_2_ photoanode after the seed layer deposition.

**Fig. 10:**
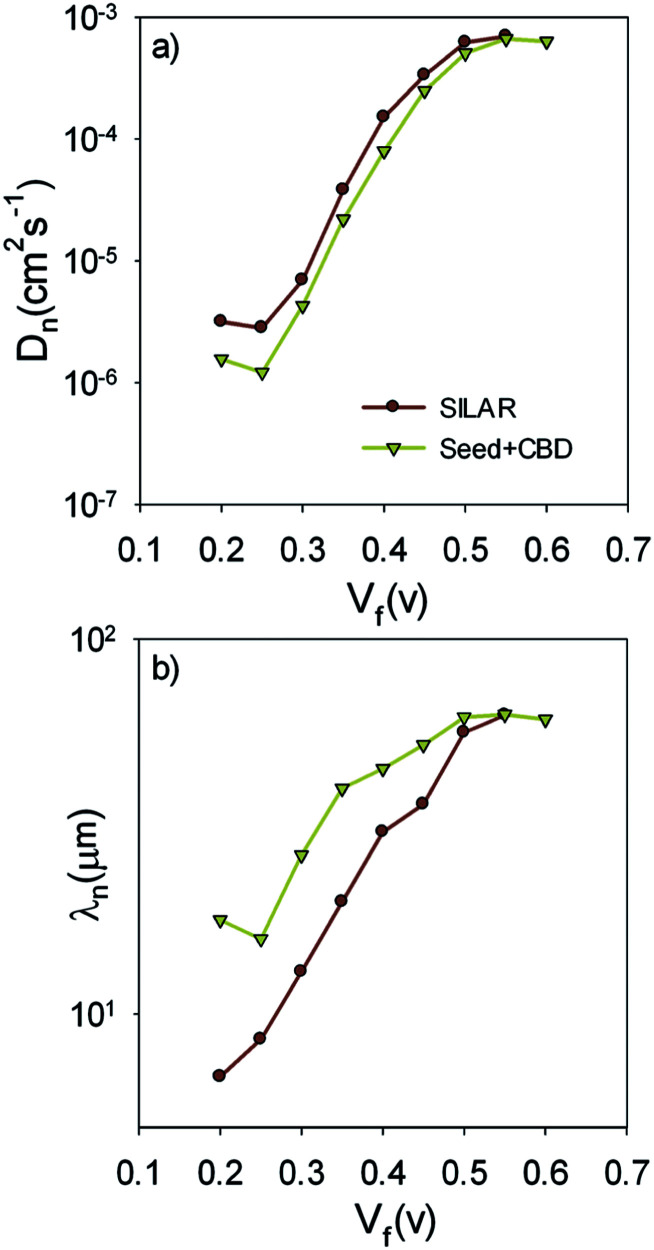
(a) Diffusion coefficient; *D*_n_, and (b) small perturbation diffusion length; *λ*_n_*versus V*_F_.

Fig. 9b presents the recombination resistance (*R*_rec_) for SILAR and Seed + CBD sensitized cells. It can be observed from this figure that seed layer deposition has enhanced recombination resistance (lower recombination rate) in compared to SILAR sensitized cells. The enhanced recombination resistance explains the origin of the higher *V*_oc_ values and fill factors^41,54^ in cells with the seed layer compared to those with SILAR sensitization (see Tables 1, S1, and S2†). This result indicates that the QDs seed layer has an effective role in reducing the recombination process in QDSCs. From the impedance results, diffusion coefficient; *D*_n_, and small perturbation diffusion length of electrons; *λ*_n_, were calculated through eqn (2) and (3), respectively,^51^ and plotted in Fig. 10a and b, respectively for S20-S400 (SILAR) and S20-S400 (Seed + CBD) structures. Here *L*, *R*_t_ and *τ*_n_ (*τ*_n_ = *R*_rec_*C*_μ_) are photoanode thickness, electron transport resistance and electron life time respectively.

2 *D*_n_ = *L*^2^(*R*_t_*C*_μ_)^−1^

3 *λ*_n_ = (*D*_n_*τ*_n_)^0.5^

According to Fig. 10a, the diffusion coefficient is reduced in cells with the seed layer. This proves the higher electron transport resistance in cells after seed layer deposition. According to eqn (3), the relative amount of the main physical parameters of cells, including chemical capacitance, recombination resistance, and transport resistance indicates the amount of the *λ*_n_ parameter. It is important to note that the effect of seed layer on *λ*_n_ was not investigated before while it has a clear insight for interpretation of the cells' performance.

Here, *λ*_n_ was markedly improved after seed layer deposition (Fig. 10b) which indicates the superior charge transport properties of the cells with the seed layer. As explained in Introduction, the seed layer is generally deposited on the photoanode structure in order to enhance the amount of QDs' sensitizer loading on the photoanode. In other words, after seed layer deposition, light absorption is enhanced and, consequently, more photogenerated electrons are expected in QDSCs. We also showed the improved light absorption after seed layer deposition according to Fig. 6a. While the effect of the seed layer on light absorption enhancement is generally accepted in the literature, our results suggest that seed layer has a crucial effect on the charge transport properties of cells, including recombination rate, diffusion coefficient, and especially diffusion lengths of electrons in cells.

**Fig. 11:**
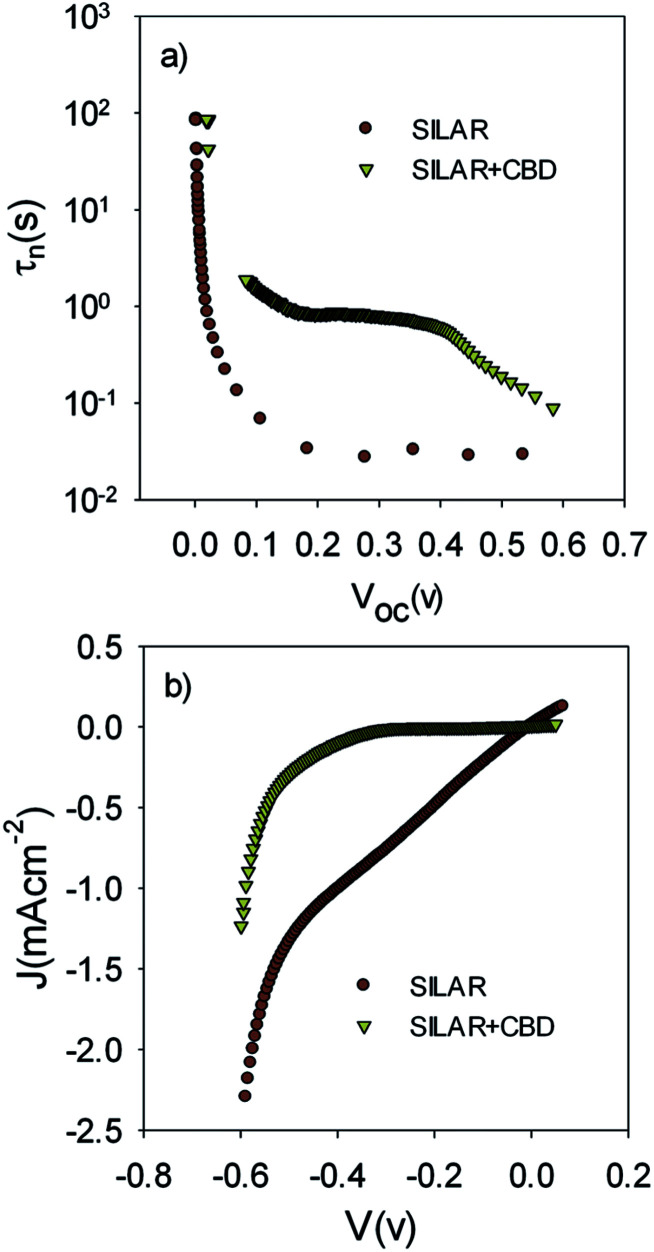
(a) Electron lifetime, *τ*_n_, of SILAR sensitized and seed layer deposited samples, *τ*_n_ has been measured by ABVD under dark conditions. (b) Current voltage characteristic of the cells in the dark condition.

The lower recombination in cells with the seed layer which was observed by IS measurements (Fig. 9b) was confirmed by comparing the electron lifetime; *τ*_n_, of S20-S400 (SILAR) and S20-S400 (Seed + CBD) cells (Fig. 11a).

**Fig. 12:**
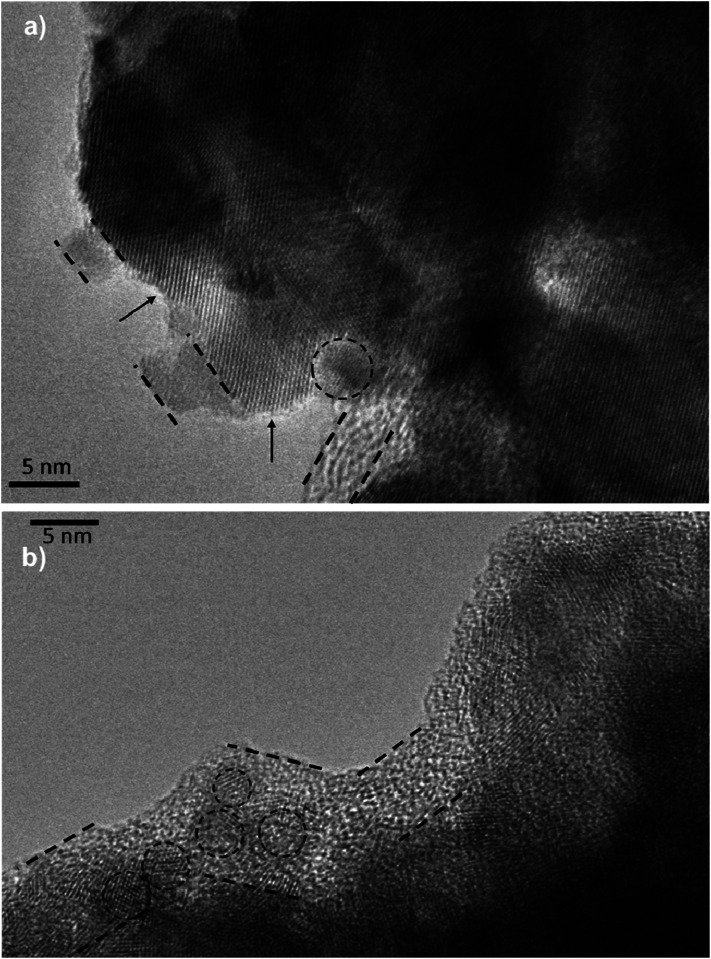
TEM micrograph from the (a) S20-S400 (SILAR) and (b) S20-S400 (Seed + CBD) samples. Some QDs are shown by dashed circles and the thin deposited layer of QDs is indicated between dashed lines. There are some places on the structure which are not well deposited by QDs and are indicated by arrows in the figure. Scale bar is 5 nm in both figures.

*τ*_n_ has been measured by ABVD^55^ method under dark conditions as IS measurements. In the case of cells with the seed layer, higher lifetimes were obtained, as expected from the higher recombination resistance previously commented on (Fig. 9b). Fig. 11b illustrates the current voltage properties of S20-S400 (SILAR) and S20-S400 (Seed + CBD) cells in the dark condition. According to this figure, lower dark currents are attained for cells with the seed layer which is in good agreement with the enhanced recombination resistance (Fig. 9b) and higher electron life time (Fig. 11a) in these cells. Here, we investigated the impedance spectroscopy result for the cells with S20-S400 structures (Fig. 4a) as it is a general structure normally used in DSSCs and QDSCs.^19,36,50^ It must be pointed out that impedance spectroscopy measurements were also performed for cells with the S200-S400 and S400-S400 structures (not shown here) and the same mechanism was observed after seed layer deposition, regardless of the type of photoanode structure. Fig. 12 presents the TEM micrograph from S20-S400 (SILAR) and S20-S400 (Seed + CBD) samples.

**Fig. 13:**
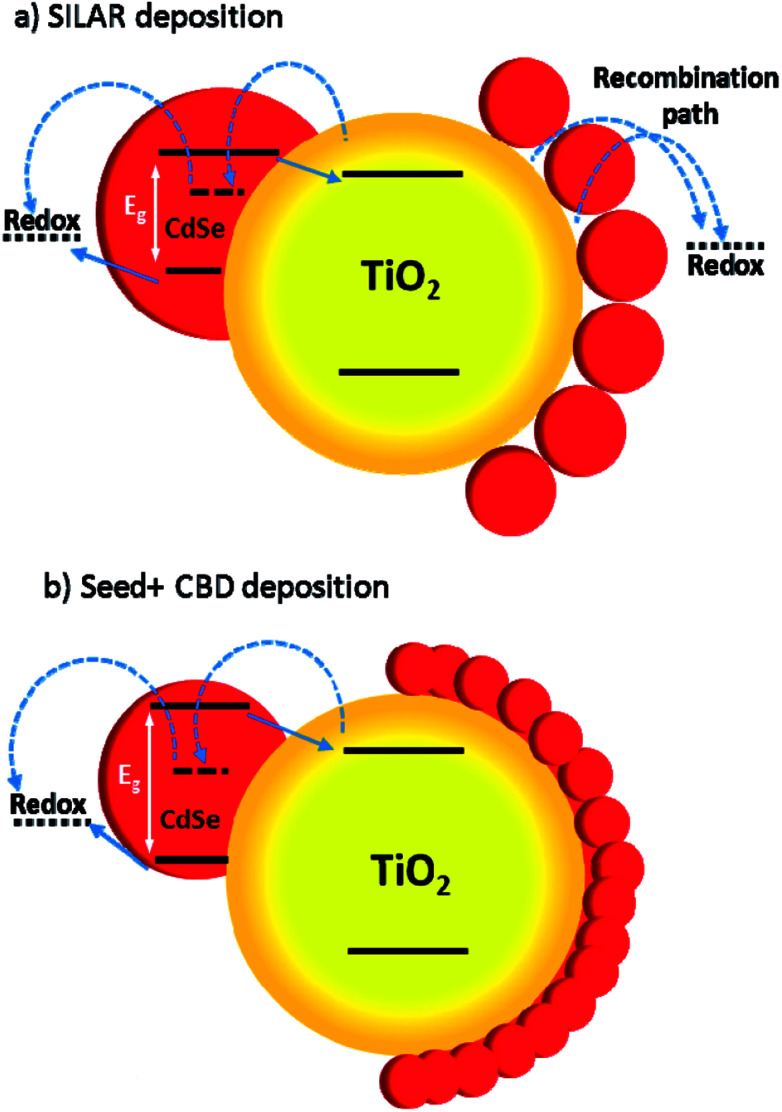
The scheme of the cells which are sensitized by SILAR (a) and Seed + CBD (b). Red circles indicate the CdSe QDs. Arrow lines indicates the favorable electron transports and dashed arrow lines indicates the recombination paths.

In Fig. 12a and b, some QDs are shown by dashed circles and the thin deposited layer of QDs is indicated between dashed lines. Based on Fig. 12a, there were some places on the structures which were not well deposited by QDs (indicated by arrows in Fig. 12), thereby enhancing the recombination. On the other hand, it seems that a more homogeneous QD layer is deposited in cells with the Seed + CBD deposition method, decreasing charge recombination in cells (Fig. 12b). With regard to the results from *J*–*V* measurements, EIS, and the TEM, the schematic structure of the cells sensitized by SILAR and Seed + CBD methods is presented in Fig. 13.

Here, red circles indicate CdSe QDs, arrow lines depict favorable electron transport paths, and dashed arrow lines show recombination paths. According to Fig. 13, CdSe QDs have larger sizes in SILAR sensitized cells than Seed + CBD sensitized ones, leading to a red shift in the absorption spectrum (Fig. 6a) and IPCE results (Fig. 5b). According to Fig. 13a, there are various areas on the TiO_2_ surface which are not covered by CdSe QDs in SILAR sensitized cells. These areas make various electron recombination paths from TiO_2_ into the redox electrolyte shown by dashed arrows on the right side of Fig. 13a. On the contrary, for Seed + CBD sensitized cells, CdSe QDs were deposited on the TiO_2_ structure in a compact structure (Fig. 13b) which reduced the electrolyte and TiO_2_ interface area. Consequently, more electron life times and lower dark currents were seen for Seed + CBD sensitized cells as explained in Fig. 11a and b respectively.

Though the system studied here, our results show new and useful insights into the mechanisms of photoinduced charge transfers in cells with seeded photoanodes. In other words while it is typically assumed that seed layer increase the light absorption,^3,33,40,41^ we approved that more complex charge transfer properties take places in the seeded photoanodes. As explained before, the seed layer increases the diffusion lengths of electrons noticeably. Consequently more thickness of TiO_2_ layer could utilized as a photoanode for further improving the conversion efficiencies, without concerning the electron recombination in cells. By increasing the photoanode thickness, deposition of QD sensitizers on the photoanode increases, therefore, enhances the light harvesting in cells. Consequently, the rate of electron–hole generation by the incident light increases and higher *V*_oc_ and *J*_sc_ values could be expected. Also, conversion efficiency of cells could improve by controlling the recombination through SiO_2_ deposition on ZnS layer as explained before.^56^

## Conclusions

We demonstrated the key role of the QD seed layer in the performance of QDSCs. The IPCE is directly affected by seed layer deposition. Higher *J*_sc_ values are obtained when SILAR is employed for various structures of the photoanode, while *V*_oc_ and fill factors are reduced. It was proved that the seed layer deposition could simply enhance the fill factor noticeably. This is systematically correlated to the higher recombination resistance (lower recombination rate) observed for the samples with the seed layer in comparison with SILAR sensitized cells, while the relative position of the TiO_2_ conduction band is not affected. It was shown that the seed layer increases the electron diffusion lengths in QDSCs. Also, the conventional view about the seed layer effect is modified here. In other words, we introduce the seed layer deposition as a method to improve the charge transport properties of the cells even in the ones with efficient light harvesting properties.

We conclude that the seed layer deposition could systematically be applied in order to optimize the QDSC performance.

## Conflicts of interest

There are no conflicts of interest to declare.

## Supplementary Material

RA-008-C8RA04413A-s001
