# Supplementary material for: Improving the parameters of electron transport in quantum dot sensitized solar cells through seed layer deposition
Source: RSC Adv. 2018 Jul 19;8(46):26056–68. doi: 10.1039/c8ra04413a (PMC9082740; doi:10.1039/c8ra04413a)
Supplement: RA-008-C8RA04413A-s001 [file RA-008-C8RA04413A-s001.pdf]

Electronic Supplementary Material (ESI) for RSC Advances.
This journal is © The Royal Society of Chemistry 2018

Supporting Information

Improving the parameters of electron transport in quantum dot sensitized solar cells through seed layer deposition

Mahmoud Samadpour,*[a]

[a] Department of Physics, K. N. Toosi University of Technology, PO Box 15418-49611, Tehran, Iran

Figure S1:

Figure S1 indicates the SEM micrograph of S20, S200, S400, S20-S400, S200-S400 and S400-S400 structures. It is important to note that the top surface of S20-S400, S200-S400 and S400-S400 structures is a S400 layer and consequently has a same morphology.

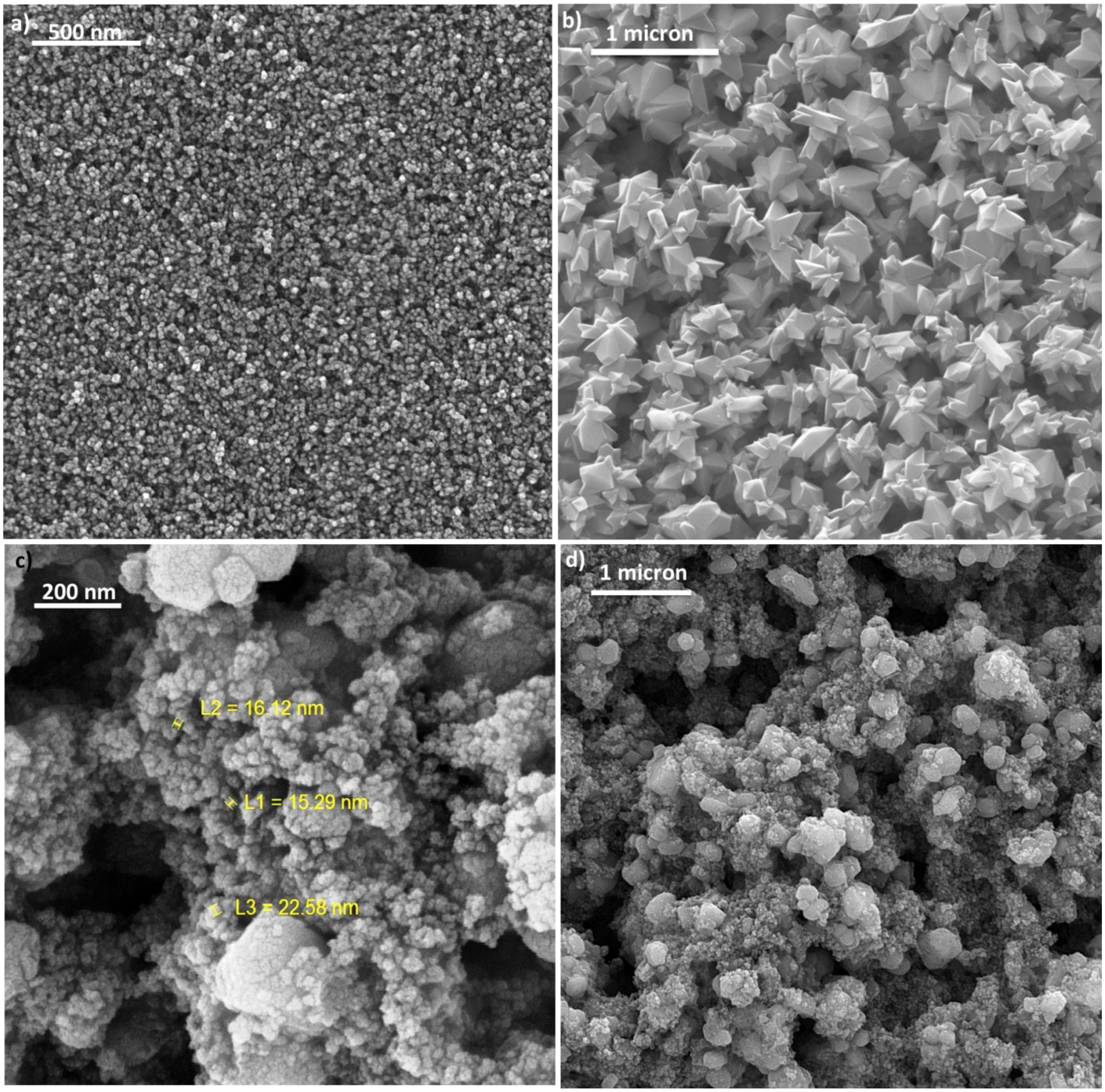

Figure S1: SEM micrograph of a) S20, b) S200, c) S400, and d) S20-S400, S200-S400 and S400-S400 structures. It is important to note that the top surface of S20-S400, S200-S400 and S400-S400 structures is a layer of S400 and consequently has a same morphology.

Figure S2:

Figure S2 presents the IPCE and current voltage properties of S200-S400 cells which were sensitized by SILAR, CBD and Seed+CBD methods. We refer to these cells as S200-S400 (SILAR), S200-S400 (CBD), and S200-S400 (Seed+CBD) for ease of discussion.

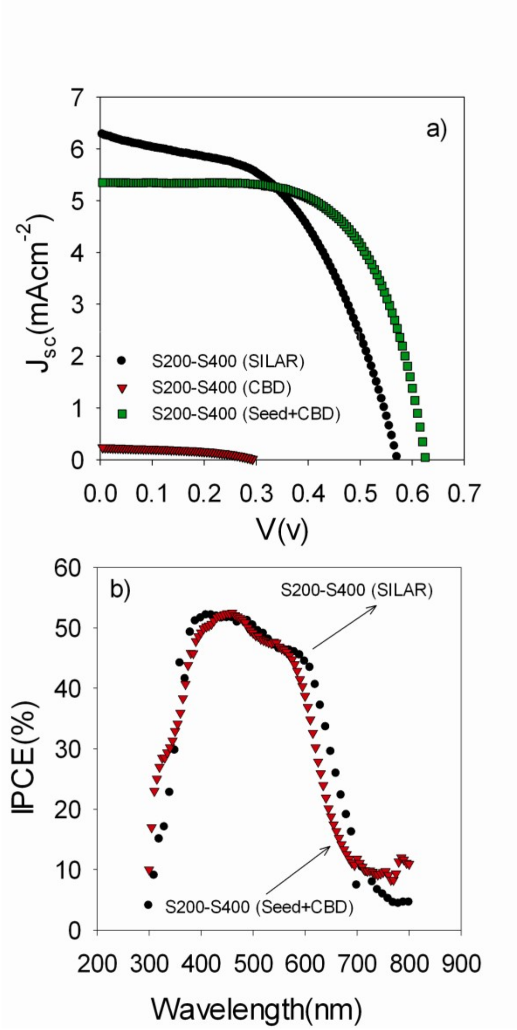

Figure S2. a) Current voltage and b) IPCE properties of the S200-S400 cells which are sensitized by SILAR, CBD and Seed+CBD method.

The corresponding photovoltaic parameters of Figure S2(a) are indicated in Table S1. From Table S1, it is clear that the CBD-sensitized cell has a very low current density (0.24 mAcm^{-2}), confirming the low QD deposition on the photoanode structure. It is important to note that the

S200 structure has a small surface area (9.86 m^2gr^{-1}) compared with S20 (71.23 m^2gr^{-1}) and S400 (29.41 m^2gr^{-1}) structures. Thus, a lower QD deposition and consequently smaller current densities are expected in these structures.

Table S1. Photovoltaic parameters of the QDSCs: photocurrent j_{sc}, open circuit voltage V_{oc}, fill factor *FF*, and efficiency *E*, as a function of the sensitization method tested under standard AM 1.5G conditions.

Anode Type	V_{oc}(mV)	J_{sc}(mA/cm^2)	FF	E (%)
S200-S400(SILAR)	570±8	6.29±0.07	0.50±0.02	1.81±0.05
S200-S400(CBD)	295±7	0.24±0.04	0.40±0.03	0.03±0.01
S200-S400(Seed+CBD)	622±6	5.24±0.06	0.61±0.01	2.00±0.03

The short circuit current density for CBD sensitized cells were too low to be measured by IPCE and is therefore not shown here (Figure S2 (b)). Based on Figure S2 (a), SILAR sensitized cells present higher current densities than CBD sensitized ones while their open circuit voltage is less. According to Figure S2(b), a clear red shift is observed in IPCE values for SILAR sensitized cells, proving that CdSe QDs have a larger size in SILAR sensitized cells in comparison to cells which are sensitized by the Seed+CBD method. According to Table S1, in spite of reduced current densities in Seed+CBD sensitized cells, open circuit voltage is enhanced which could be related to the reduced recombination rate of electrons from the photoanode into the redox electrolyte, upward shift of the TiO_2 conduction band, or improved charge transport properties in cells which is investigated in more detail in the main text.

Figure S3: Figure S3 presents the IPCE and current voltage properties of S400-S400 cells which were sensitized by SILAR, CBD and Seed+CBD methods. We refer to these cells as S400-S400 (SILAR), S2400-S400 (CBD), and S400-S400 (Seed+CBD) for ease of discussion.

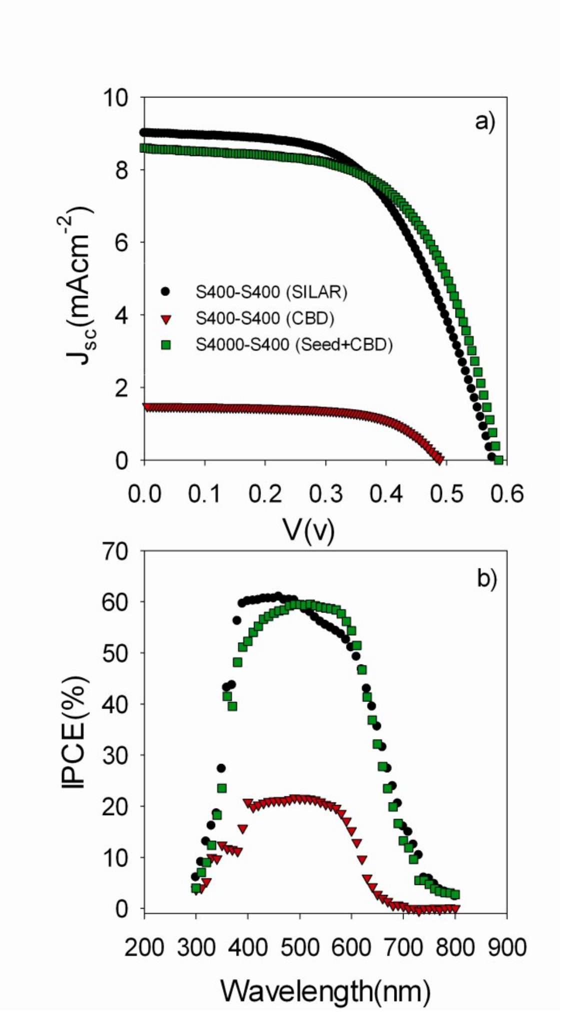

Figure S3. a) Current voltage and b) IPCE properties of the S400-S400 cells which are sensitized by SILAR, CBD and SILAR (3 cycles of CdSe as a seed layer)/CBD (Seed+CBD) method.

The corresponding photovoltaic parameters of Figure S3(a) are presented in Table S2.

Table S2. Photovoltaic parameters of the QDSCs: photocurrent j_{sc}, open circuit voltage V_{oc}, fill factor *FF*, and efficiency *E*, as a function of the sensitization method tested under standard AM 1.5G conditions.

Anode Type	V_{oc}(mV)	J_{sc}(mA/cm^2)	FF	E (%)
S400-S400(SILAR)	581±3	9.01±0.04	0.55±0.01	2.90±0.03
S400-S400(CBD)	490±7	1.52±0.08	0.62±0.01	0.46±0.05
S400-S400(Seed+CBD)	590±2	8.63±0.06	0.60±0.01	3.08±0.02

From Table S2, it is obvious that CBD sensitized cells have higher current densities (1.52 mAcm^{-2}) than CBD sensitized S200-S400 ones (Table S1, 0.24 mAcm^{-2}) which is related to the enhanced surface area of S400-S400 cells in comparison to S200-S400 cells. Furthermore, the current density in CBD sensitized cells is still much lower than that of cells which are sensitized by SILAR or Seed+CBD methods (Table S2). According to Figure S3(a), SILAR sensitized cells present higher current densities than CBD sensitized ones, while their open circuit voltage and fill factor is lower.
